# Development of ZIF-Derived Nanoporous Carbon and Cobalt Sulfide-Based Electrode Material for Supercapacitor

**DOI:** 10.3390/ma12182940

**Published:** 2019-09-11

**Authors:** Rabia Ahmad, Naseem Iqbal, Tayyaba Noor

**Affiliations:** 1 US–Pakistan Center for Advanced Studies in Energy (USPCAS–E), National University of Sciences and Technology (NUST), Islamabad 44000, Pakistan; 2 School of Chemical and Materials Engineering (SCME), National University of Sciences and Technology (NUST), Islamabad 44000, Pakistan

**Keywords:** ZIF-67, water, methanol, sulfidation, specific capacitance

## Abstract

Zeolitic Imidazolate Framework (ZIF-67) was prepared in two different solvents—water and methanol. Nanoporous carbon was derived from ZIF-67 via pyrolysis in an inert atmosphere. Anion exchange step of sulfidation on the synthesized material has a great influence on the structure and properties. Structural morphology and thermal stability were characterized by X-ray diffraction (XRD), scanning electron microscopy (SEM)/energy dispersive x-ray spectroscopy (EDS), Brunauer-Emmett-Teller (BET), and thermogravimetric (TG) analysis. The electrochemical analysis was evaluated by cyclic voltammetry, chronopotentiometry, and impedance analysis. The as-prepared nanoporous carbon and cobalt sulfide (NPC/CS) electrode material (water) in 2M KOH electrolyte solution exhibit high specific capacitance of 677 F/g. The excellent electrochemical performance of the NPC/CS was attributed to its hierarchical structure. This functionalized ZIF driven strategy paves the way to the preparation of various metal oxide and metal sulfide-based nanoheterostructures by varying the type of metal.

## 1. Introduction

With the affluence of the energy industry, several countries have paid much attention to the advancement of power sources. Presently, studies on supercapacitors are rare, compared to the energy storage devices of batteries. As an alternative energy source, supercapacitors have the advantages of high power density, long cyclic stability, and low-cost [1,2,3]. The main four parts of a supercapacitor are electrode material, electrolyte, separator, and collector. Electrode material exhibits a primary role in the performance of the supercapacitors [4,5].

Among the reported electrode materials for the supercapacitors, metallic oxides and metallic hydroxides have very good theoretical specific capacity but they have very poor electrical conductivity and poor cyclic stability [6]. At present, metallic sulfides have gained many attractions for the applications in supercapacitors as they have very good specific capacitance and electrical conductivity, supporting the improved electrochemical features [7]. Metalic sulfides electrode materials have a high reversible Faradic reaction at the electrode/electrolyte interface that occurs during the charge transfer process. A number of sulfides use supercapacitor applications, i.e., MnS [8], CuS [9], MoS [10], CoS [11], NiS [12], as well as bimetallic sulfides such as NiCo_2_S_4_ [7]; although, the enhancement in surface area, porosity, and mechanical support can improve the electrochemical properties of the metallic sulfides. For that purpose, graphene/graphene oxide and metallic sulfide-based composed like NiS/reduced GO, Co_3_S_4_ growth on graphene have been reported as a promising electrode material for the applications in supercapacitors [11].

The metal-organic frameworks (MOFs) have deployed a great sway in the development of supercapacitors since the MOFs were formed in the late 1990s [13]. Excessive consideration was paid to ZIF-67 just because of its polyhedral framework. Due to the number of porosities, considerable surface area, small density, thermal, and chemical stabilities [1], ZIFs represent a breakthrough in the various applications comprising adsorption/separation [14,15], sensors [16], catalysis [17], gas storage [18], and drug delivery [19].

Metal-organic framework (MOF) (and by extension, ZIFs) is a class of nanoporous material that is assembled by coordinated bonds between the two main components—metal ions and organic linker—to shape a 3D porous assembly [4]. Tremendous porosity and surface area, exceptional pore size, and chemical permanency, in ZIFs like ZIF-8 and ZIF-67, are extensively useful in numerous applications such as gas storage [2], separation technologies [14], catalysis [16], and energy-related fields [1]. ZIFs with particle-like morphology remained dominant in use so far, and it has been an eye-catching task to control their framework. It is commonly believed that different structural evaluation stages, like the first formation of nucleation, initiation crystallization, and then growth, are tangled in the crystallization of ZIFs [2]. In recent times, a new 2D leaf-like structure was prepared by using metal ions of zinc and the linker 2–methylimidazole. It is well acknowledged that morphology and particle size have a great effect on both the extensive and intensive characteristics of the material [20,21]. ZIFs derived nanoporous carbon (NPC) and metal oxide (MO) based material has a synergistic effect of both carbon-based material and metallic oxide. Furthermore, in comparison with the metal oxide-based material, metal sulfide-based electrode materials have superior electrocatalytic activity [22,23].

In the present work, we synthesized cobalt sulfide onto the nanoporous carbon to positively incorporate the synergistic effect towards the electrical conductivity and stability in two different solvents. Here we demonstrated, the 2D leaf-like morphology exhibits the superior capacity and the best electrochemical performance. Effect of solvent i.e., water and methanol, on the synthesis of ZIF-67-derived nanoporous carbon and cobalt sulfide-based electrode, and then measured specific capacitance, is also investigated for the application in supercapacitor.

## 2. Materials and Methods

### 2.1. Chemicals

Metal ion used is cobalt nitrate hexahydrate (Co(NO_3_)_2_·6H_2_O, 99%), and the linker is 2–Methylimidazole (99%) were used. All the chemicals were purchased from Sigma Aldrich/Merck, have analytical purity and used as received.

### 2.2. Synthesis of ZIF-67

For the synthesis of ZIF-67, the following scheme was used; 0.873 g of cobalt nitrate hexahydrate was dissolved in 30 mL of methanol to form a clear solution; 0.984 g of organic linker 2–Methylimidazole was dissolved in 10 mL of methanol to make another clear solution. The two solutions mixed with a vigorous shake of a few minutes. The mixed solution was kept overnight at room temperature. Thenceforth, centrifugation is used to collect the precipitates followed by multiple washes using methanol and dried up at 80 °C for 6 hours. The same experimental procedure was used to synthesis ZIF-67 using deionized water.

### 2.3. Preparation of Nanoporous Carbon (NPC) and Cobalt Oxide (CO)

The dried powder ZIF-67 particles were heated at 350 °C for 1.5 hours, raised to 700 °C at a ramp rate of 4 °C per minute, followed by pyrolysis for 3.5 hours under a flowing argon atmosphere. Next, the prepared black fluffy powder was cooled to room temperature naturally.

### 2.4. Preparation of Nanoporous Carbon (NPC) and Cobalt Sulfide (CS)

Aqueous suspension of nanoporous carbon-containing cobalt oxide was stirred in 0.015M sodium sulfide for 30 minutes, and then solution mixture transferred to a stainless steel autoclave of Teflon-lined and heated at 120 °C for 6 hours. As obtained precipitates washed and dried.

### 2.5. Material Characterization

The surface morphological analysis of the polyhedral structure was described by field emission scanning electron microscopy (VEGA3, 51–ADD0007) (Tescan, Brno, Czech Republic). The identification of a crystalline structure was elucidated by X-ray diffraction on a diffractometer (D8 Advance, CuKR, λ = 1.54Å) (Bruker, Karlsruhe, Germany). The thermogravimetric analysis was conducted on a DTG–60H (Shimadzu, Kyoto, Japan) instrument in the temperature range of room temperature to 800 °C. The surface areas and porous structure of synthesized NPC/CO and NPC/CS were measured by Brunauer–Emmett–Teller analysis using NovaWin 20e (Quantachrome, Virginia, USA) instrument at a relative pressure p/p^o^ = 0–1.0 and the samples were degassed at 160 °C under the vacuum.

### 2.6. Electrochemical Testing

The electrochemical measurements of the prepared samples were performed on an electrochemical workstation CHI 760E (CH Instrument, Texas, USA) with a setup of three electrodes. A reference electrode Ag/AgCl (SC) and a counter electrode of a platinum coil are used. To prepare the ink for the working electrode (GC), the following scheme was used; catalyst (2 mg) was dispersed ultrasonically for 1 to 2 hours in 0.08 mL of ethanol solution with 0.02 mL of Nafion solution (5 wt. %) to form a homogeneous ink. Then, the polished glassy carbon electrode (3 mm diameter) was coated by dropping the suspension (5 µL).

Electrochemical impedance spectroscopy (EIS) used a frequency field of 1 to 100 kHz in 2 M KOH solution. Cyclic voltammetry was performed within the potential window of 0.5 V in 2 M KOH solution with various sweep rates of 10, 20, 50, 80, and 100 mV s^−1^. The chronopotentiometry technique is used to measure the charge-discharge curve at 0.01 mA cathodic current to obtain the discharge time in the potential window of 0.35 V.

## 3. Results

### 3.1. Morphology/Structural Analysis 

**Figure 1:**
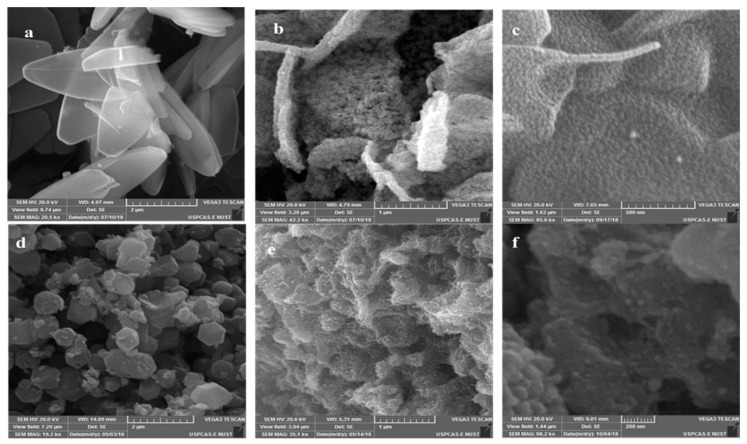
SEM images of ZIF-67 prepared in different solvents: (**a**) ZIF-67 prepared in H_2_O, before pyrolysis, after pyrolysis; (**b**) NPC/CO (H_2_O) at 700 °C; (**c**) NPC/CS (H_2_O); (**d**) ZIF-67 prepared in MeOH; (**e**) NPC/CO (MeOH) at 700 °C; and (**f**) NPC/CS (MeOH).

The framework and surface morphology of the as-prepared specimen were explored on SEM (Figure 1). Metal-organic framework (MOF) ZIF-67 displayed a different framework of structure with a different solvent. ZIF-67 prepared in methanol exhibits well-defined polyhedrons with a smooth surface. The shape of ZIF-67 showed typical and uniform rhombic dodecahedron assembly, which is consistent with the morphology of ZIF-67 in the literature [4]. The synthesized product with water as a solvent (Figure 1a) showed 2D leaf-like morphology, and the side length of this leaf-like morphology is approximately 2.498 micron. In Figure 1d, in the rhombic face, the side length is ~430 nm. The appearance of each rhombic face is smooth, proposing high purity of the as-prepared product ZIF-67. Furthermore, the sharp edges and clear-cut corners of ZIF-67 particles (see Figure 1d) demonstrate the ascertaining of the crystallography characteristics.

**Figure 2:**
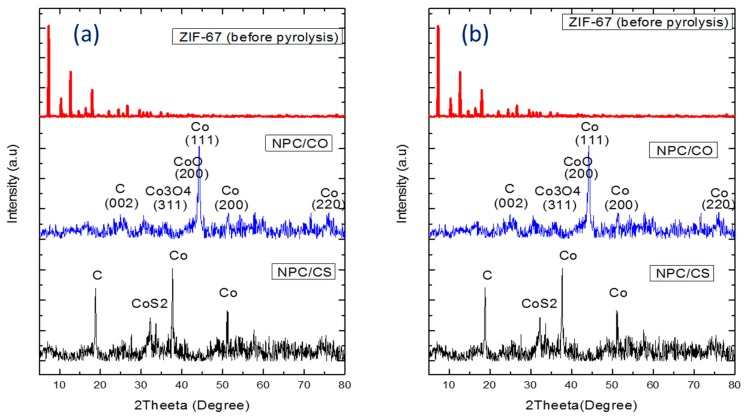
XRD patterns of (**a**) ZIF-67, NPC/CO and NPC/CS prepared in H2O and (**b**) ZIF-67, NPC/CO, and NPC/CS prepared in MeOH.

The crystal structure of ZIF-67 before and after pyrolysis was examined by XRD measurements. The relative intensity and peak positions are well-matched with the literature [24], which shows that ZIF-67 was successfully synthesized. The wide diffraction peak at 2θ =25° belongs to peak (002) of graphite carbon. The diffraction peaks derived for the metallic cobalt at the (111) phase and (200) phase were detected at 2θ= 45° and 52° (JCPDS card No. 15–0806). All diffraction peaks of nanoporous carbon and cobalt sulfide, as shown in Figure 2, correspond well with the patterns reported in the previous study [25,26]. Through the sulfidation treatment, CoO particles were successfully transformed into the well-defined CoS_2_ cubic phase (JCPDS card No. 41–1471).

**Figure 3:**
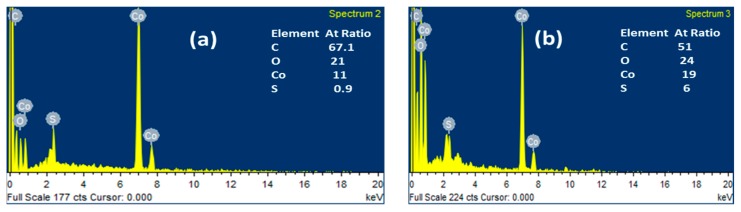
EDS spectrum of (**a**) NPC/CS (H2O) and (**b**) NPC/CS (MeOH).

The atomic ratio of C: Co:O:S is confirmed by the energy-dispersive X-ray spectroscopy (EDS) analyses (Figure 3).

**Figure 4:**
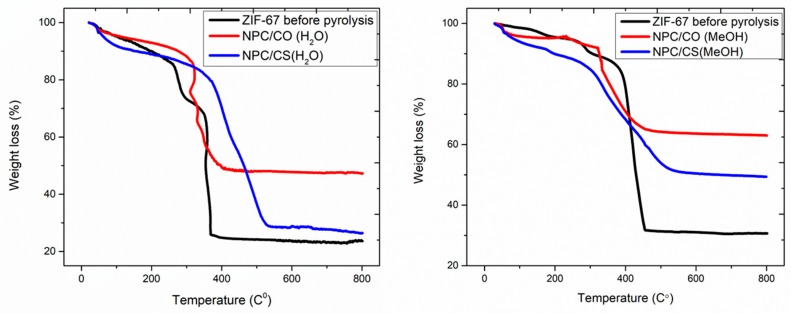
TGA curve analysis of as prepared ZIF-67, NPC/CO, and NPC/CS.

TGA curves of prepared ZIF-67, nanoporous carbon/cobalt oxide (NPC/CO), and nanoporous carbon/cobalt sulfide (NPC/CS) under flowing nitrogen condition, as shown in Figure 4. The first stage mass loss of approximately 11%, below 200 °C, and a 6% loss from 200 °C to 320 °C correspond to the removal of guest water molecules, i.e., surface moisture, solvent, nitrates, and weekly bounded linker molecules, respectively [27]. Next, the rapid degradation occurred with the mass losses from 320 °C in H_2_O-based system and 316°C MeOH-based system. Finally, no further weight loss was observed after 530°C of NPC/CS (H2O) and NPC/CS (MeOH), the quasistatic point, and the residual mass was comprised of metal oxide [28].

In the thermal analysis, Figure 4 curves indicate that nanoporous carbon and cobalt sulfide doped samples have high thermal stability (Table 1).

The N_2_ adsorption-desorption isotherms for as-prepared samples, which is characterized as type I and type II hysteresis loops according to the IUPAC classification, indicating the microporous characteristics of the synthesized sample. Nitrogen adsorption can be considered as the first stage in the characterization of microporous and mesoporous solids. In the vision of the complexity of the condensation and evaporation mechanisms, one should not be supposed to be able to conclude a reliable pore size distribution unless certain conditions are met. It is recommended that the shape and location of the hysteresis loop should always be taken into account before any computation [29].

**Figure 5:**
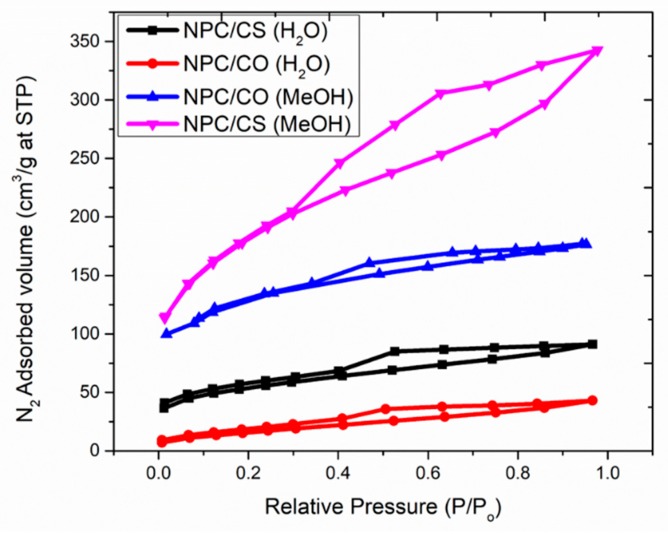
N_2_ adsorption isotherms at 77 K.

It can be identified that the specific surface areas of NPC/CS have increased largely with the treatment of sulfidation, as the analysis is presented in Table 2. From the Figure 5, it can be clearly seen Type I isotherm is given by all the materials except NPC/CS (MeOH). Type I isotherms are microporous materials that acquire mostly wider micropores and possibly narrow mesopores. In the case of NPC/CS (MeOH), type II isotherm materials are often disordered and the distribution of pore size and shape is not well-defined [30]. The type II isotherm is the consequence of the open monolayer-to-multilayer adsorption up to p/p_0_.

The enhanced surface area can be attributed to smaller diameters and larger quantities of the nanocrystals and nanopores. The total pore volume of NPC/CS showed an increment as compared to parent NPC/CO.

### 3.2. Electrochemical Testing

The measurement and investigation of electrochemical behavior were explored by galvanostatic charge-discharge (GCD), cyclic voltammetry (CV), and electrochemical impedance spectroscopy (EIS) in 2M KOH solution.

By definition, capacitance = charge/voltage [31].

Capacitance value can be calculated from resulting cyclic voltammogram using the following equation [32].

(1) $$C=\frac{Q}{2\Delta Vm}$$

where *C* is the specific capacitance in (Figure 1), *Q* is the average integral area under the curve, ∆*V* is the potential window in volts, and m is the mass loading of the active material in the working electrode (g).

The energy density (ED) uses the relation [32]:

(2) $$ED=\frac{1}{2}\cdot C\frac{{\left(V\right)}^{2}}{3.6}$$

where *C* is the value of specific capacitance and V is the voltage window.

The reversible process was observed in all cases. The linear trend across the whole range of scan rates reveals that the process is reversible.

**Figure 6:**
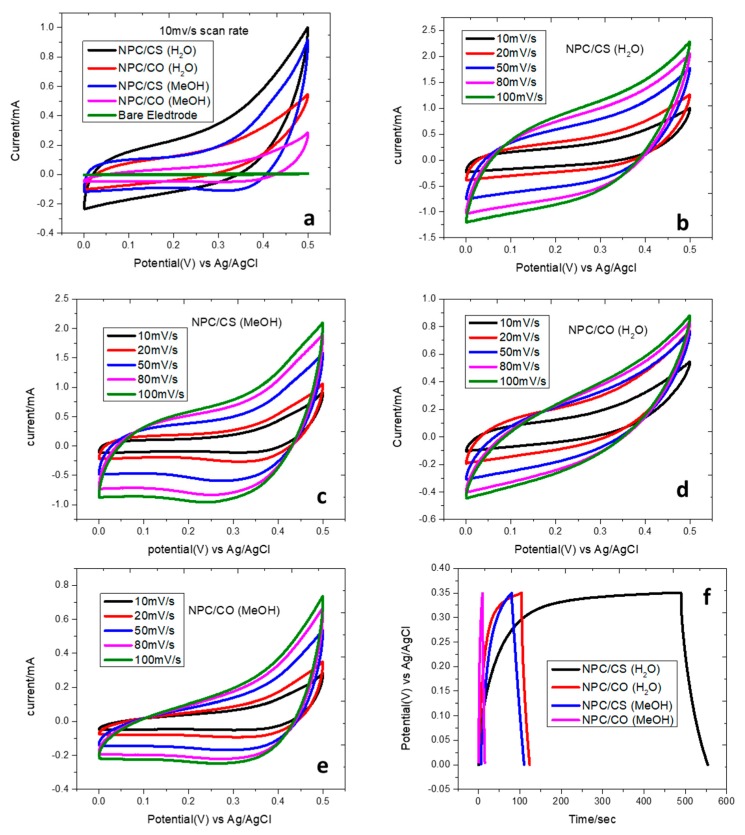
Electrochemical properties of the NPC/CO and NPC/CS in 2M KOH: (**a**) cyclic voltammetry at 10 mv/s scan rate (**b**–**e**) at different scan rates and (**f**) charge-discharge profiles at 0.01 mA/g.

The electrochemical performance of ZIF-67-derived NPC/CO and NPC/CS were studied by varying the scan rate ranging from 10mV/s to 100 mV/s CV curves and shown in Figure 6. The anodic peaks and cathodic peaks, which are related to positive current and negative current, respectively, in the CV curves, originate from the oxidation and reduction process of the cobalt cation, which indicates that the capacitance aspects are primarily driven by Faradaic redox reaction.

It was observed that as the scan rate increases from 10mV/s to 100mV/s, the specific capacitance increases. This variation in capacitance reveals that at low scan rate, inner parts and outer part of the nanoporous material exhibited the redox reaction, whereas, for high scan rate, the only outer part of the material involved redox reaction [33,34]. There are interfaces faces present in the case of oxides that causes the limitation in the connectivity or the flow of electrons, while this is reported by many of authors that the deposition of sulfide on oxide containing composite reduces these interfaces and produce connectivity in the flow. This will contribute to enhancing conductivity [11,35]. Thus the specific capacitance and the energy density calculated is given in Table 3.

### 3.3. Cyclic Stability Study

**Figure 7:**
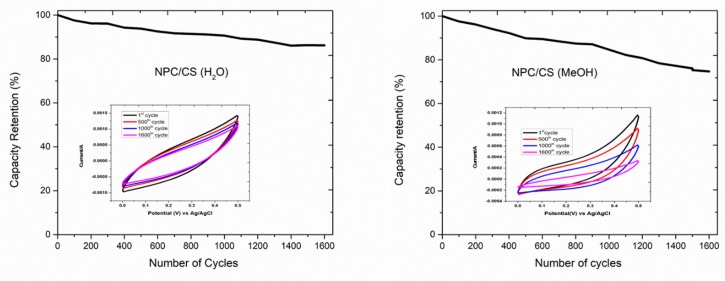
Numbers of cycles versus capacitive retention for the electrode NPC/CS electrode materials recorded over 1600 cycles at a scan rate of 100 mV/s.

One of the most essential and considerable parts in the achievement of a supercapacitor is cyclic stability. Figure 7 shows the electrochemical performance of the nanoporous carbon; the cobalt sulfide-based electrode recorded over 1600 cycles with a scan rate of 100 mV/s. The cyclic stability study demonstrates the change in the specific capacitance of the NPC/CS electrode with the number of cycles. The specific capacitance decreases with 86% retention capacity over 1600 cycles for the case of NPC/CS (H_2_O) and noted as 74% over 1600 cycles for the NPC/CS (MeOH) systems. Zhu et al. [36] presented nearly 74% stability aimed at the nickel sulfide electrode material by recording 1000 cycles in a potassium hydroxide electrolyte; a nearly identical method to that of the cinnamon-like electrode. This decomposition was perceived in cycling stability. This might be expected because of the disintegration of the active electrode material in the electrolyte and the capacity imbalances between the electrochemical electrodes, which originates the instability of the electrode potential [37].

### 3.4. Electrochemical Impedance Spectroscopy Study

**Figure 8:**
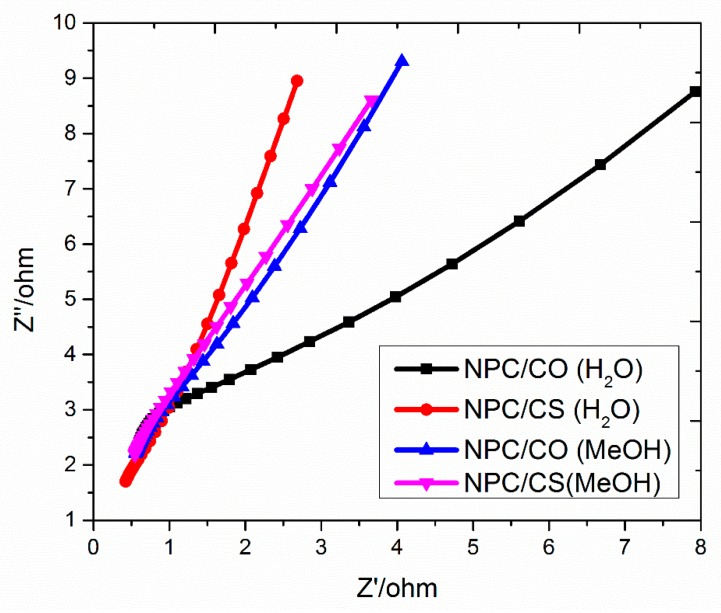
Nyquist plot of the NPC/CO and NPC/CS.

To evaluate the frequency performance and confrontation attitude of any material, electrochemical impedance spectroscopy is a robust engine [38]. The EIS spectra are recorded in a frequency ranging from 100 kHz to 1 Hz and shown in Figure 8. The perfect supercapacitor has a greater slop in a low-frequency region, which signifies electro–capacitive behavior [39,40].

It can be seen from Figure 8 that the smaller arc diameter in the EIS spectrum of nanoporous carbon and cobalt sulfide-based materials showed lower charge transfer resistance and more idealistic properties as compared to nanoporous carbon and metal oxide electrodes [41]. The slop of NPC/CS (water) approach has ideally straight line, inferring the superior accessibility of ions.

## 4. Conclusions

In summary, we have successfully fabricated ZIF-67-derived nanoporous carbon and metal sulfide-based electrode material in a simple and economical method. SEM and XRD confirmed the synthesis of nanoporous material with increased surface area and 2D morphology. The enhanced electrochemical performance, due to sulfidation of cobalt oxide, was investigated by CV GCD and EIS. The specific capacitance value has increased to 677 F/g in the case of NPC/CS (H_2_O). A novel and facile route for the synthesis of nanoporous and binder-free electrode material is proposed with increased specific capacitance for the energy storage application.

## Figures and Tables

**Table 1:** TGA thermal analysis of ZIF-derived nanoporous carbon and cobalt oxide/sulfide-based materials.

Compound	Temp. Range (°C)	Mass Loss or Residue (%)
ZIF-67 (MeOH) before pyrolysis ZIF-67 (H_2_O) before pyrolysis	336–458 302–373	57 48
NPC/CO (MeOH) NPC/CO (H_2_O)	314–492 297–414	28 40
NPC/CS (MeOH) NPC/CS (H_2_O)	300–544 311–535	34 56

**Table 2:** Surface area, pore volume, and average pore size of NPC/CO and NPC/CS.

Electrode Material	Surface Area (m^2^/g)	Pore Volume (cm^3^/g)	Average Pore Size (nm)
NPC/CO (MeOH)	726.3	0.273	1.277
NPC/CS (MeOH)	934.5	0.532	1.677
NPC/CO (H_2_O)	264.6	0.063	1.544
NPC/CS (H_2_O)	521.4	0.141	2.25

**Table 3:** Specific capacitance calculated from cyclic voltammetry.

Electrode Material	Specific Capacitance (F/g)	Energy Density (Wh/kg)
NPC/CO (MeOH)	159	5.520
NPC/CS (MeOH)	480	16.666
NPC/CS (H_2_O)	373	12.951
NPC/CS (H_2_O)	677	23.506
